# A novel prediction score determining individual clinical outcome 3 months after juvenile stroke (PREDICT-score)

**DOI:** 10.1007/s00415-024-12552-5

**Published:** 2024-07-31

**Authors:** Verena S. Hoffmann, Sonja Schönecker, Moustafa Amin, Paul Reidler, Anna Brauer, Anna Kopczak, Silke Wunderlich, Sven Poli, Katharina Althaus, Susanne Müller, Ulrich Mansmann, Lars Kellert

**Affiliations:** 1 Institute for Medical Information Processing, Biometry and Epidemiology, Faculty of Medicine, Ludwig-Maximilians University München, Marchioninistr. 15, 81377 Munich, Germany; 2 Department of Neurology, LMU University Hospital, Ludwig-Maximilians-Universität München, Munich, Germany; 3 Department of Radiology, Medical Faculty, Ludwig-Maximilians-Universität München, Munich, Germany; 4 Institute for Stroke and Dementia Research (ISD), Medical Faculty, Ludwig-Maximilians-Universität München, Munich, Germany; 5 Department of Neurology, University Hospital Rechts der Isar of the Technical University Munich, Munich, Germany; 6 Department of Neurology & Stroke, Hertie Institute for Clinical Brain Research, Eberhard-Karls-University Tübingen, Tübingen, Germany; 7 Department of Neurology, University of Ulm, Ulm, Germany; 8 Pettenkofer School for Public Health, Munich, Germany

**Keywords:** Prediction score, 3-month outcome, Juvenile stroke, Predictive factors

## Abstract

**Background:**

Juvenile strokes (< 55 years) account for about 15% of all ischemic strokes. Structured data on clinical outcome in those patients are sparse. Here, we aimed to fill this gap by systematically collecting relevant data and modeling a juvenile stroke prediction score for the 3-month functional outcome.

**Methods:**

We retrospectively integrated and analyzed clinical and outcome data of juvenile stroke and TIA patients treated at the LMU University Hospital, LMU Munich, Munich. Good outcome was defined as a modified Rankin Scale of 0–2 or return to baseline of function. We analyzed candidate predictors and developed a predictive model. Predictive abilities were inspected using Area Under the ROC curve (AUROC) and visual representation of the calibration. The model was validated internally.

**Results:**

346 patients were included in the analysis. We observed a good outcome in *n* = 293 patients (84.7%). The prediction model for an unfavourable outcome had an AUROC of 89.1% (95% CI 83.3–93.1%). The model includes age NIHSS, ASPECTS, blood glucose and type of vessel occlusion as predictors for the individual patient outcome.

**Conclusions:**

Here, we introduce the highly accurate PREDICT-score for the 3-month outcome after juvenile stroke derived from clinical routine data. The PREDICT-score might be helpful in guiding individual patient decisions and designing future studies but needs further prospective validation which is already planned.

*Trial registration* The study has been registered at https://drks.de (DRKS00024407) on March 31, 2022.

## Introduction

Stroke is one of the leading causes of death and permanent disability worldwide, accounting for approximately 6.5 million deaths worldwide and approximately 140 disability adjusted life years [1]. It is primarily a disease of the elderly, although around 15% occur in people under the age of 55. In addition, it is precisely in this age group that the incidence has increased by up to 40% in recent years [2, 3].

The etiology of juvenile stroke usually differs from that of older patients. It is particularly challenging that the etiology is much more diverse and for many cases (up to 30%) etiology remains unknown [2].

Despite the low prevalence of strokes at a younger age, the individual and socioeconomic consequences are very significant due to the even longer lifespan [4, 5].

For this reason, it is essential to determine predictors of outcome after juvenile strokes. To date, there are no studies dedicated to the outcome of juvenile strokes. Validated clinical parameters can enable personalized decisions and lay the foundation for future clinical trials. This study aims to address this gap by modeling a multivariable juvenile stroke prediction score for functional outcome at 3 months after stroke, using a combined set of clinical and paraclinical data.

## Materials and methods

### Ethics statement

Ethical approval for retrospective analysis of data has been obtained at the local ethics committee at LMU Munich (21-0136). The study is conducted according to the Declaration of Helsinki.

### Study design and patients

We retrospectively collected clinical, imaging and laboratory data in juvenile stroke and transient ischemic attack (TIA) patients who were hospitalized at the stroke unit of the LMU University Hospital, LMU Munich, Munich, Germany between Jan 01, 2011 and Mar 31, 2020. Study size was defined by the number of patients treated during this period. Data were extracted from the clinical database by trained personnel and integrated into our study database.

Ischemic stroke was defined by a sudden focal neurologic deficit lasting more than 24 h with no sign of acute intracranial bleeding on cerebral imaging at admission. TIA was defined as a brief episode of focal loss of brain function that lasted less than 24 h, thought to be due to ischemia, localized to a region of the brain supplied by one vascular system and for which no other cause could be found [6]. Trained stroke neurologists performed physical and neurological examinations on admission and treated patients according to current guidelines for the management of stroke during their in-hospital stay. Reperfusion therapy by intravenous thrombolysis with a recombinant tissue plasminogen activator, mechanical thrombectomy or both was performed as appropriate.

### Selection of candidate predictors

A systematic literature review was conducted to identify potential predictor variables of functional outcome in juvenile stroke or transient ischemic attack (TIA). We selected for further consideration variables that are collected as part of clinical routine in the majority of stroke patients and have been reported to be associated with poor outcome. The relevant variables are based on expert opinion of the authors and are the well-known predicting variables in stroke care.

Variables were divided into four categories. The first consisted of *preadmission factors*, including age, previous stroke or TIA as well as the time from symptom onset to admission. The second category comprised *clinical, imaging, and laboratory findings* at admission, including clinical severity measured by the National Institutes of Health Stroke Scale score (NIHSS), systolic blood pressure, blood glucose level as well as the Alberta stroke program early CT score (ASPECTS) or the posterior circulation ASPECTS (pc-ASPECTS) and large vessel occlusion (LVO). LVO was defined as proximal artery occlusion suitable for thrombectomy. The third category included the *results of diagnostic investigations during the in-hospital stay*, like mean carotid artery intima–media thickness (IMT) on ultrasonography, the presence and severity of a patent foramen ovale [PFO, examined in a transesophageal echocardiogram (TEE)], CHA_2_DS_2_-VASc-Score and atrial septal aneurysm (ASA), respectively, and the underlying aetiology. The fourth category consisted of the *treatment* given, including intravenous thrombolysis with a recombinant tissue plasminogen activator and vessel occlusion measured by the modified Thrombolysis in Cerebral Infarction (mTICI) score.

Age, CHA_2_DS_2_-VASc-Score, time from symptom onset to admission, NIHSS score, blood pressure, glucose level, ASPECTS and mean IMT were analyzed as continuous variables while the variable previous stroke or TIA was dichotomized. As the aetiology of juvenile stroke is more heterogeneous compared to older stroke patients, in addition to the underlying Trial of Org 10172 in Acute Stroke Treatment (TOAST) mechanisms [7] we included the presence of a cervical artery dissection, moyamoya disease and vasculitis as independent aetiologies in our data collection.

### Outcome

All stroke patients were asked to participate in a clinical structured follow-up 3 months after stroke. Trained personnel assessed the 3-month functional outcome either during an outpatient visit or via a structured follow-up telephone interview. They were blinded with respect to clinical data during the in-hospital stay. A favorable outcome at 3 months was defined as a modified Rankin Scale (mRS) of 0–2 or return to baseline of pre-stroke function. Higher values on the mRS were deemed unfavorable outcomes.

### Statistical analysis

Patient characteristics, clinical parameters and outcomes were analyzed descriptively using total numbers and percentages or median and Interquartile ranges (IQR). Univariate Odds Ratios with 95% CIs for an unfavourable outcome were calculated using univariate logistic regression. *P* values of the respective Wald tests were also added.

### Missing data

Patients with missing outcome information were deleted from the data set. Missing observations for candidate predictors were imputed five times with the Multiple Imputation by Chained Equations (MICE) algorithm using all other predictors in the data set as well as the outcome and the random forest method in the R package mice [8]. Random Forest imputation is known for its robustness and ability to handle complex interactions and nonlinear relationships in the data [9]. Imputations were used for multivariate modeling but not for the univariate analysis, e.g., shown in Table 1.

**Table 1:** Baseline characteristics and outcome of patients in total numbers (%) or median and interquartile ranges (IQR)

	Total (*n* = 346) *n* (%) or median (IQR)	Missing *n* (%)	OR (95% CI)	*P* value
Outcome: modified Ranking Scale at 3 months		–		
0	118 (34.1%)			
1	113 (32.7%)			
2	62 (17.9%)			
3	21 (6.1%)			
4	16 (4.6%)			
5	5 (1.4%)			
6	11 (3.2%)			
Model outcome		–		
Favourable (mRS 0–2)	293 (84.7%)			
Unfavourable (mRS 3–6)	53 (15.3%)			
*Preadmission factors*				
Age (years)	49 (42–53)	0	1.10 (1.04–1.16)	0.0009
Previous stroke or TIA	70 (20.3%)	1 (< 1%)	0.59 (0.30–1.14)	0.1179
CHA_2_DS_2_-VASc-score		0		
2	84 (24.3%)		Reference	
3	169 (48.8%)		0.84 (0.38–1.86)	0.6680
4	81 (23.4%)		1.90 (0.83–4.32)	0.1273
5	10 (2.9%)		4.42 (1.07–18.21)	0.0394
6	2 (< 1%)		6.64 (0.39–113.98)	0.1920
Time from symptom onset to admission (h)	9.1 (2.3–26.1)		1.00 (0.99–1.00)	0.2704
*Clinical, imaging and laboratory findings at admission*				
NIHSS	2 (1–6)	3 (< 1%)	1.18 (1.12–1.23)	< 0.0001
Blood pressure (systolic mm/Hg)	151 (140–168)	128 (37%)	1.00 (0.98–1.01)	0.471
Glucose mg/dl	108.0 (96.0–127.5)	8 (2%)	1.02 (1.01–2.02)	< 0.0001
ASPECTS	10 (9–10)	42 (12%)	0.61 (0.51–0.73)	< 0.0001
*Results of diagnostic investigations during the in-hospital stay*				
Mean IMT (mm)	0.6 (0.53–0.72)	73 (21%)	36.61 (3.96–338.42)	0.0015
Presence and severity of PFO		0		
No PFO/small PFO without ASA	172 (49.7%)		Reference	
Relevant PFO	49 (14.2%)		0.58 (0.19–1.75)	0.3310
No TEE performed	125 (36.1%)		1.70 (0.92–3.15)	0.0908
Etiology		0		
Large artery atherosclerosis	29 (8.4%)		Reference	
Small vessel diseases	11 (3.2%)		0.14 (0.02–1.26)	0.0795
Cervical artery dissection	38 (11.0%)		0.32 (0.11–0.96)	0.0430
Atrial fibrillation	18 (5.2%)		0.28 (0.07–1.20)	0.0868
Other cardioembolic causes	23 (6.6%)		0.50 (0.15–1.64)	0.2530
Other etiology	34 (9.8%)		0.29 (0.10–0.84)	0.0224
Cryptogenic	180 (52.0%)		0.14 (0.06–0.34)	< 0.0001
Primary CNS vasculitis	13 (3.8%)			
*Treatment*				
Intravenous thrombolysis		2 (< 1%)		
Thrombolysis performed	93 (27%)		Reference	
No thrombolysis and NIHSS ≤ 2	157 (45.6%)		0.09 (0.03–0.27)	< 0.0001
No thrombolysis and NIHSS > 2	94 (27.3%)		1.38 (0.71–2.67)	0.3372
Vessel occlusion		9 (2.6%)		
No vessel occlusion detectable on CTA	200 (59.3%)		Reference	
Distal vessel occlusion	23 (6.8%)		2.58 (0.66–10.01)	0.1716
Large vessel occlusion but no mechanical thrombectomy performed or TICI < 2B	62 (18.4%)		8.18 (3.65–18.36)	< 0.0001
Large vessel occlusion and TICI 2B or 3	52 (15.4%)		8.35 (3.60–19.33)	< 0.0001

Univariate odds ratios with 95% CIs for an unfavourable outcome. *P* values of the respective Wald tests

### Model development

As prior research has shown that machine learning models are not superior to regression analysis [10–12] multivariate logistic regression analyses were performed to assess the association of candidate predictors with the 3-month functional outcome endpoint.

To derive an appropriate prediction model we created all possible models on each of the five imputation data sets using the R package glmulti [13]. We then chose the variables with a model-averaged importance of terms of over 0.8 for further modeling using Akaike’s Information Criterion (AIC) and variable selection in each imputation data set.

Linearity of the relationship between the log (Odds Ratio) and the continuous predictors were checked graphically. Outliers and influential observations were identified using Cook’s distance and standardized residuals. Collinearity was assessed by calculating the Variance Inflation Factor, goodness of fit was evaluated via the Hosmer–Lemeshow test.

To pool the models from each imputation data set to achieve one final model we used the extended Median-P-Rule which performs very well also when categorical variables are used [14]. This method is included in the R package psfmi [15].

### Predictive ability and validation

Model discrimination was visualized by plotting the ROC curve and calculating the Area Under the ROC curve (AUROC) with corresponding DeLong 95% confidence intervals (CI). Calibration was assessed by plotting the mean observed probability against the mean predicted probability in each decile. Perfect calibration is displayed as a straight line passing through zero with a gradient of one.

The model was validated internally by performing a bootstrap validation of the final model using 1000 bootstrap samples to achieve an optimism corrected AUROCC.

All analyses were performed using R version 4.3.1.

### Data sharing

The data of this study are available on site from the corresponding author upon reasonable request.

This work is reported according to the suggestions made in the TRIPOD statement [16].

## Results

**Fig. 1:**
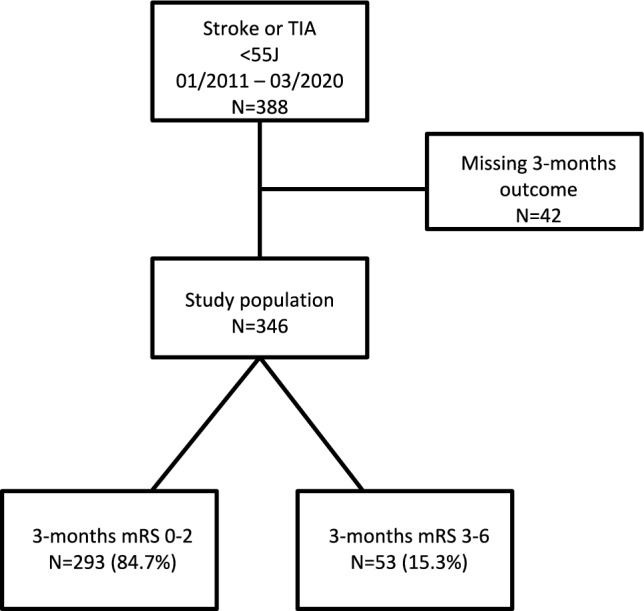
Flow of patients through the study and outcome status

From Jan 01, 2011 to Mar 31, 2020 the inclusion criteria of juvenile stroke or TIA were met by 388 consecutive patients treated at the Department of Neurology of the LMU University Hospital. Data of these patients were collected from clinical routine documentation. For 42 patients the 3-month outcome was not available. These observations were excluded and the data of 346 patients were included into the final analysis (see Fig. 1). Table 1 shows the baseline characteristics of the final cohort and the univariate associations of the candidate predictors with the patient outcome 3 months after stroke or TIA as Odds Ratios (OR), the 95% confidence intervals (95% CI) and the *P* values of the respective Wald tests. Age, NIHSS, glucose level, ASPECTS, mean IMT, etiology, intravenous thrombolysis and vessel occlusion were significant predictors in the univariate analysis.

### Missing data

Data were missing mainly for the candidate predictors systolic blood pressure at admission (*n* = 128, 37%), mean IMT (*n* = 73, 21%) and ASPECTS (*n* = 42, 12%). Unfortunately, systolic blood pressure was not systematically recorded from 2011 through 2014, thus it is missing more frequently than other values. Systolic blood pressure was missing significantly more frequently in patients with a favourable outcome (40.3% vs. 18.9%, *P* = 0.0049), while mean IMT was missing significantly more often in patients with an unfavourable outcome (16.0% vs. 49.1%, *P* < 0.0001). There was no significant difference between missing values for ASPECTS between patients with a favourable and an unfavourable outcome (11.3% vs. 17.0%, *P* = 0.3449). All missing values were imputed using the MICE algorithm.

### Multivariate analysis

In the multivariate logistic regression analyses the variables vessel occlusion, NIHSS, ASPECTS, and blood glucose level were the variables with a model-averaged importance of terms of over 0.8 in each imputation data set. The variable age had a model-averaged importance of terms over 0.8 in four out of the five imputation data sets.

The AIC of the models including age additionally to vessel occlusion, NIHSS, ASPECTS, and blood glucose level had lower AICs in each of the five imputation data sets, thus we included age into the final model. In each imputation data set, the continuous predictors age and blood glucose level were assessed for their functional form using plots of the observed log odds versus predictor value. The linearity assumption was not violated. Absolute values of standardized residuals were never larger than three indicating that no single observation had an overly high impact on the model’s fit. A sensitivity analysis excluding five outliers with a Cook’s distance of more than 0.04 did not result in different predictors or changed model coefficient estimates. There were no indicators for overdispersion or collinearity. The Hosmer–Lemeshow goodness-of-fit test was not significant.

**Fig. 2:**
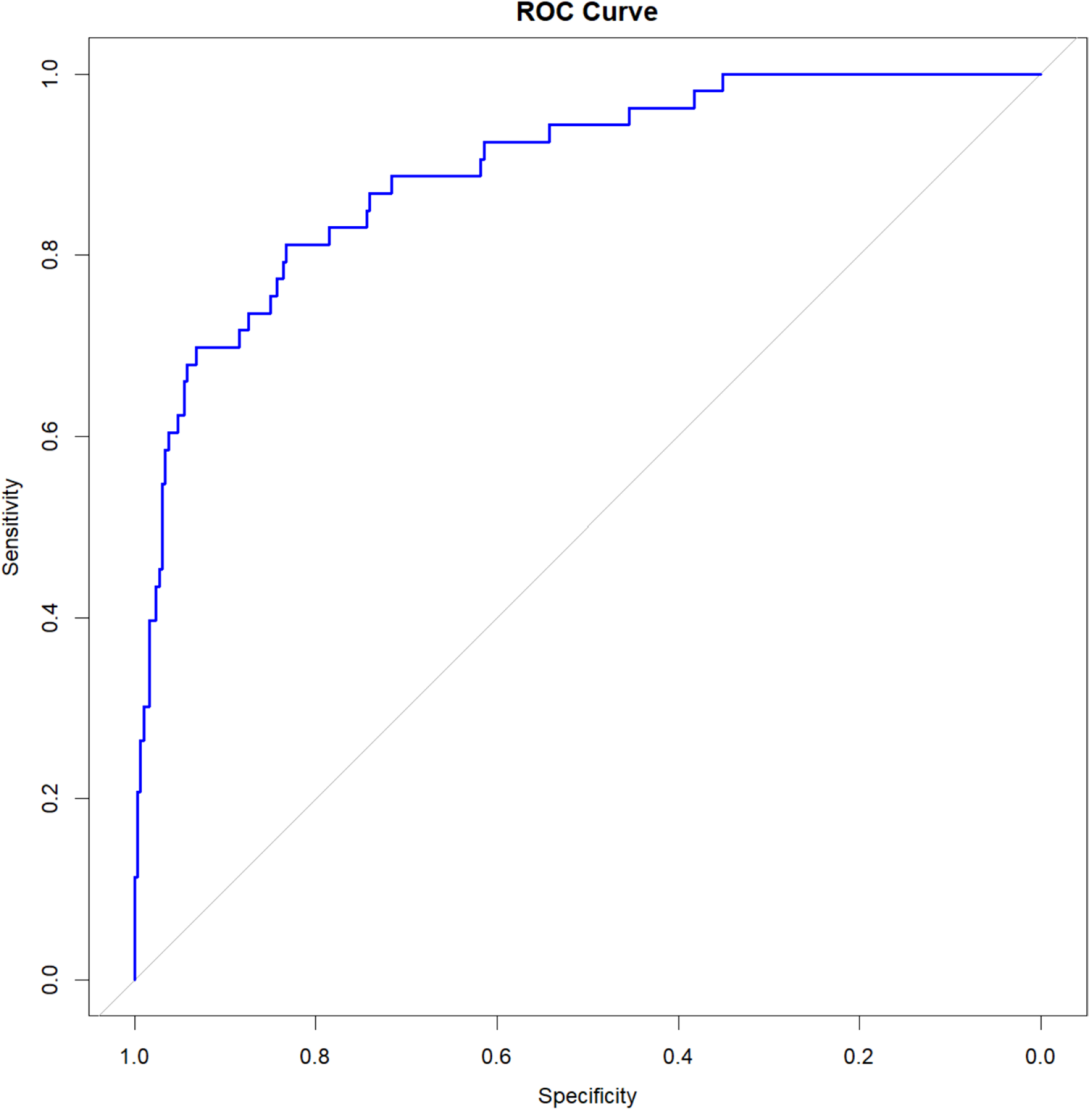
Blue line represents the ROC curve, the grey line represents the ROC curve of an uninformative model

**Fig. 3:**
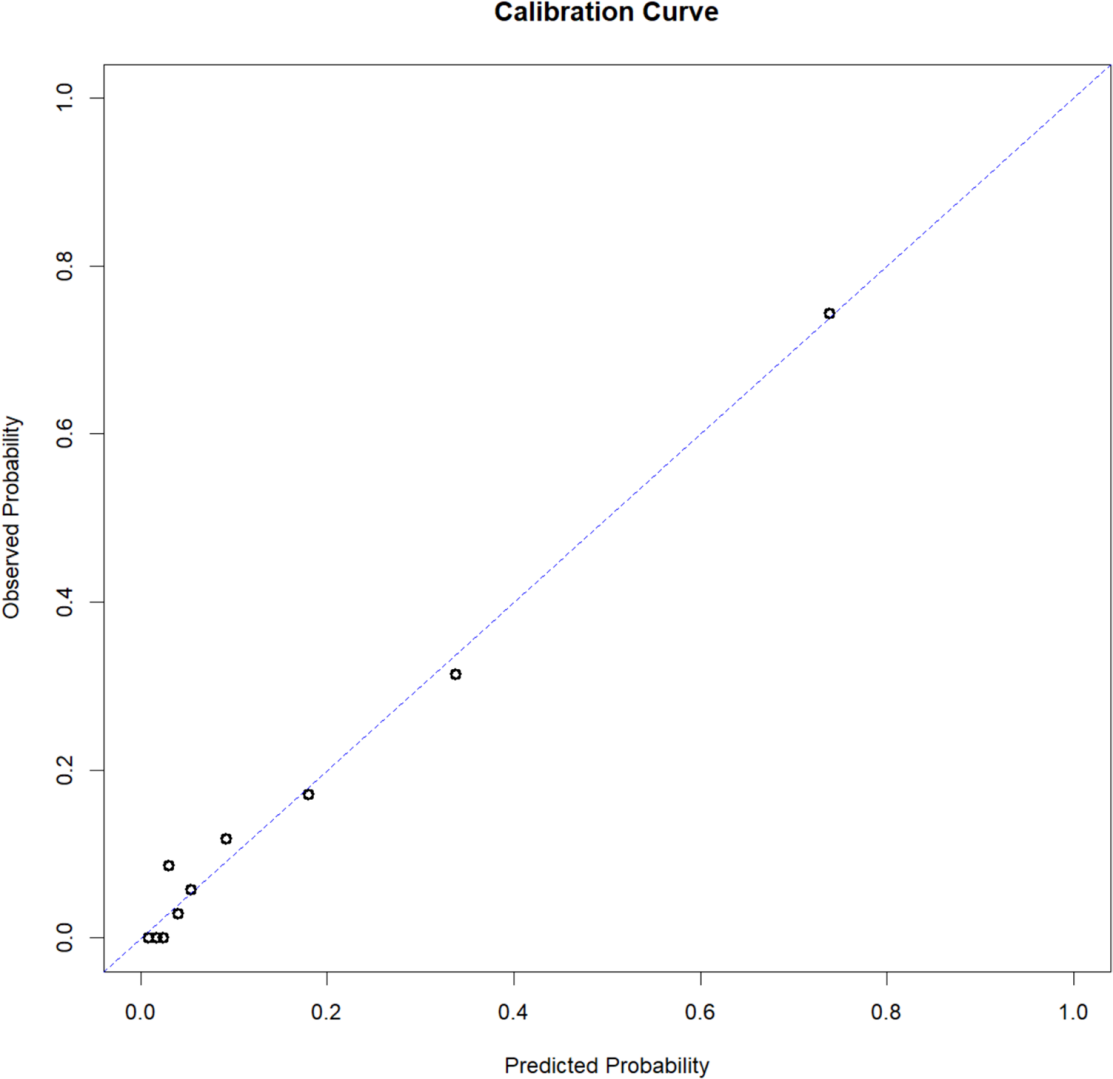
Calibration plot: graphical representation of the predicted probability of an unfavourable outcome against the actual probability of an unfavourable outcome. Patients were ranked into order of predicted probability of an unfavourable outcome and divided into tenths. The dots represent the mean risks for each tenth; the dotted line represents the perfect relationship

In a final step, we pooled the model with the five predictors vessel occlusion, NIHSS, ASPECTS, blood glucose level and age. The model estimates, ORs and p values are given in Table 2. The AUROC of the model is 89.1% (95% CI 83.3–93.1%). The ROC curve (Fig. 2) shows the model’s very good discrimination. The calibration plot indicates the model is well calibrated with an intercept of 0.0009 and a slope of 0.994 (Fig. 3).

**Table 2:** Final multivariate logistic regression model for outcome 3 months after stroke

	Parameter estimate	Standard error	OR (95% CI)	*P* value
Age (years)	0.07347	0.0333	1.08 (1.01–1.15)	0.0283
NIHSS	0.1223	0.0284	1.13 (1.07–1.19)	< 0.0001
ASPECTS	− 0.2740	0.1160	0.76 (0.61–0.95)	0.0192
Glucose mg/dl	0.0147	0.0037	1.01 (1.01–1.02)	< 0.0001
Vessel occlusion				
No vessel occlusion detectable on CTA	Reference			
Distal vessel occlusion	− 0.5996	1.0167	0.55 (0.07–4.03)	0.5578
Large vessel occlusion but no mechanical thrombectomy performed or TICI < 2B	1.4444	0.5270	4.24 (1.51–11.91)	0.0068
Large vessel occlusion and TICI 2B or 3	0.1739	0.6046	1.19 (0.36–3.89)	0.7739

The internal validation via bootstrapping resulted in an optimism corrected AUROC of 87.5%.

The score for this model can be calculated for the individual patient as:

PREDICT Score = − 6.1265 + 0.07347 * age + 0.1223 * NIHSS − 0.2740 * ASPECTS + 0.0147 * glucose mg/dl − 0.5996 * distal vessel occlusion (yes = 1, no = 0) + 1.4444 * large vessel occlusion but no mechanical thrombectomy performed or TICI < 2B (yes = 1, no = 0) + 0.1739 * large vessel occlusion and TICI 2B or 3 (yes = 1, no = 0).

The individual predicted probability for an unfavourable outcome can be assessed by calculating exp(PREDICT Score)/1 − exp(PREDICT Score). However, the resulting probability needs to be interpreted keeping the low overall percentage of 15.3% of patients with an unfavourable outcome in mind.

## Discussion

Up to now, to our knowledge there is no tool predicting outcomes especially for juvenile stroke patients. This represents a significant gap in patient care and clinical research as especially younger patients need a valid prediction to adjust their family and work circumstances if needed. The PREDICT score presented in this work is very precisely predicting the outcome of juvenile stroke after 3 months using the mRS with a cutoff at 0–2 for favorable outcomes in our cohort. The mRS is the most frequently used primary outcome measure in acute ischemic stroke and the used cutoff is well established [17], thus the PREDICT score can help to guide further decisions regarding potentially complicated clinical procedures, planning for special rehabilitation facilities and designing clinical trials.

For calculation of the PREDICT score no additional data besides clinical routine parameters are required. All relevant score parameters are available within the first hours of hyperacute stroke care allowing a fast prediction, thus the score has the potential to become part of the clinical routine in the treatment of juvenile stroke.

One limitation of our work is the retrospective collection of data from a single center. Recalibration of the score might be indicated in different settings depending on the ratio of unfavourable outcomes in the individual hospital/care unit. In addition, the data were collected from an almost 10-year period (Jan 01, 2011 to Mar 31, 2020). The rather long time period was necessary to reach a minimum number of unfavourable outcomes—which are rather rare in juvenile stroke patients—for stable model estimation. Therefore, we cannot rule out that changes in stroke treatment over time affected the outcome. Although patient data was well documented we had to impute substantial parts for three candidate predictors [systolic blood pressure (*n* = 128, 37%), mean IMT (*n* = 73, 21%) and ASPECTS (*n* = 42, 12%)].

The major limitation of our work is the absence of a validation in an independent cohort. However, data for an external and temporal validation will be collected from routine care data in a structured manner in our institution as well as our partner institutions. The protocol of this validation study has already been published [18]. In this data we will also be able to do more subgroup analyses, for example for age groups and etiology.

Presence and severity of a PFO might be a predictor of interest in further research. In our data we observed a non-significant but potentially substantial protective effect of a relevant PFO compared to no PFO/small PFO without ASA [OR: 0.58 (95% CI 0.19–1.75)] which appears counterintuitive. However, in our cohort patients who were not examined with a TEE did have an increased risk to experience an unfavourable outcome [OR: 1.70 (95% CI 0.92–3.15)]. This can in part be explained because TEE was not regularly performed when the cause of stroke was already known. It would be interesting to know if even with a known cause for stroke PFO might be an independent predictor of the outcome.

The selected predictors in the PREDICT score appear plausible as they were found to be predictive in earlier research on functional outcome after stroke, e.g. age, NIHSS and glucose [10, 19, 20]. Systolic blood pressure, however, was found to be predictive in earlier research but not in our data [10, 21]. This might be due to the high percentage of missing values (37%) or to lower relevance of this predictor for younger patients. We hope to clarify this matter using data from the planned validation cohort.

The PREDICT-scores’ accuracy in our patient cohort is comparable or even better than e.g. recent prediction models for elderly patients based on MRI imaging and clinical deep learning model reaching an AUROC of 90% and 68% [10, 11, 22].

## Conclusion

Here we introduce the highly accurate PREDICT-score for 3-month outcome after juvenile stroke derived from clinical routine data. The PREDICT-score might be helpful in guiding individual patient decisions and designing future studies but needs further prospective validation.

## References

[1] Feigin VL, Forouzanfar MH, Krishnamurthi R, Mensah GA, Connor M, Bennett DA et al (2014) Global and regional burden of stroke during 1990–2010: findings from the global burden of disease study 2010. Lancet 383(9913):245–254. 10.1016/s0140-6736(13)61953-4 24449944 10.1016/s0140-6736(13)61953-4 PMC4181600

[2] Béjot Y, Delpont B, Giroud M (2016) Rising stroke incidence in young adults: more epidemiological evidence, more questions to be answered. J Am Heart Assoc 5(5):e003661. 10.1161/jaha.116.003661 27169549 10.1161/jaha.116.003661 PMC4889213

[3] Griffiths D, Sturm J (2011) Epidemiology and etiology of young stroke. Stroke Res Treat 2011:209370. 10.4061/2011/209370 21789269 10.4061/2011/209370 PMC3140048

[4] Maaijwee NA, Rutten-Jacobs LC, Schaapsmeerders P, van Dijk EJ, de Leeuw FE (2014) Ischaemic stroke in young adults: risk factors and long-term consequences. Nat Rev Neurol 10(6):315–325. 10.1038/nrneurol.2014.72 24776923 10.1038/nrneurol.2014.72

[5] Maaijwee NA, Rutten-Jacobs LC, Arntz RM, Schaapsmeerders P, Schoonderwaldt HC, van Dijk EJ et al (2014) Long-term increased risk of unemployment after young stroke: a long-term follow-up study. Neurology 83(13):1132–1138. 10.1212/wnl.0000000000000817 25128177 10.1212/wnl.0000000000000817

[6] National Institute of Neurological Disorders and Stroke (1990) Classification of cerebrovascular diseases III. Stroke 21(4):637–676. 10.1161/01.STR.21.4.637 2326846 10.1161/01.STR.21.4.637

[7] Adams HP Jr, Bendixen BH, Kappelle LJ, Biller J, Love BB, Gordon DL et al (1993) Classification of subtype of acute ischemic stroke. Definitions for use in a multicenter clinical trial. TOAST. Trial of Org 10172 in acute stroke treatment. Stroke 24(1):35–41. 10.1161/01.str.24.1.35 7678184 10.1161/01.str.24.1.35

[8] van Buuren S, Groothuis-Oudshoorn K (2011) mice: multivariate imputation by chained equations in R. J Stat Softw 45(3):1–67. 10.18637/jss.v045.i03 10.18637/jss.v045.i03

[9] Shah AD, Bartlett JW, Carpenter J, Nicholas O, Hemingway H (2014) Comparison of random forest and parametric imputation models for imputing missing data using MICE: a CALIBER study. Am J Epidemiol 179(6):764–774. 10.1093/aje/kwt312 24589914 10.1093/aje/kwt312 PMC3939843

[10] Alaka SA, Menon BK, Brobbey A, Williamson T, Goyal M, Demchuk AM et al (2020) Functional outcome prediction in ischemic stroke: a comparison of machine learning algorithms and regression models. Front Neurol 11:889. 10.3389/fneur.2020.00889 32982920 10.3389/fneur.2020.00889 PMC7479334

[11] van Os HJA, Ramos LA, Hilbert A, van Leeuwen M, van Walderveen MAA, Kruyt ND et al (2018) Predicting outcome of endovascular treatment for acute ischemic stroke: potential value of machine learning algorithms. Front Neurol 9:784. 10.3389/fneur.2018.00784 30319525 10.3389/fneur.2018.00784 PMC6167479

[12] Wang W, Kiik M, Peek N, Curcin V, Marshall IJ, Rudd AG et al (2020) A systematic review of machine learning models for predicting outcomes of stroke with structured data. PLoS ONE 15(6):e0234722. 10.1371/journal.pone.0234722 32530947 10.1371/journal.pone.0234722 PMC7292406

[13] Calcagno V, de Mazancourt C (2010) glmulti: an R package for easy automated model selection with (generalized) linear models. J Stat Softw 34(12):1–29. 10.18637/jss.v034.i12 10.18637/jss.v034.i12

[14] Panken AM, Heymans MW (2022) A simple pooling method for variable selection in multiply imputed datasets outperformed complex methods. BMC Med Res Methodol 22(1):214. 10.1186/s12874-022-01693-8 35927610 10.1186/s12874-022-01693-8 PMC9351113

[15] Heymans M (2023) psfmi: prediction model pooling, selection and performance evaluation across multiply imputed datasets. https://mwheymans.github.io/psfmi/

[16] Collins GS, Reitsma JB, Altman DG, Moons KG (2015) Transparent reporting of a multivariable prediction model for individual prognosis or diagnosis (TRIPOD): the TRIPOD statement. J Clin Epidemiol 68(2):134–143. 10.1016/j.jclinepi.2014.11.010 25579640 10.1016/j.jclinepi.2014.11.010

[17] Goyal M, Ospel JM, Kappelhof M, Ganesh A (2021) Challenges of outcome prediction for acute stroke treatment decisions. Stroke 52(5):1921–1928. 10.1161/STROKEAHA.120.033785 33765866 10.1161/STROKEAHA.120.033785

[18] Schönecker S, Hoffmann V, Albashiti F, Thasler R, Hagedorn M, Louiset M-L et al (2023) PREDICT-juvenile-stroke: PRospective evaluation of a prediction score determining individual clinical outcome three months after ischemic stroke in young adults—a study protocol. BMC Neurol 23(1):2. 10.1186/s12883-022-03003-7 36597038 10.1186/s12883-022-03003-7 PMC9811707

[19] Campagnini S, Arienti C, Patrini M, Liuzzi P, Mannini A, Carrozza MC (2022) Machine learning methods for functional recovery prediction and prognosis in post-stroke rehabilitation: a systematic review. J Neuroeng Rehabil 19(1):54. 10.1186/s12984-022-01032-4 35659246 10.1186/s12984-022-01032-4 PMC9166382

[20] Fernandez-Lozano C, Hervella P, Mato-Abad V, Rodríguez-Yáñez M, Suárez-Garaboa S, López-Dequidt I et al (2021) Random forest-based prediction of stroke outcome. Sci Rep 11(1):10071. 10.1038/s41598-021-89434-7 33980906 10.1038/s41598-021-89434-7 PMC8115135

[21] Gkantzios A, Kokkotis C, Tsiptsios D, Moustakidis S, Gkartzonika E, Avramidis T et al (2023) Evaluation of blood biomarkers and parameters for the prediction of stroke survivors’ functional outcome upon discharge utilizing explainable machine learning. Diagnostics 13(3):532 36766637 10.3390/diagnostics13030532 PMC9914778

[22] Liu Y, Yu Y, Ouyang J, Jiang B, Yang G, Ostmeier S et al (2023) Functional outcome prediction in acute ischemic stroke using a fused imaging and clinical deep learning model. Stroke 54(9):2316–2327. 10.1161/STROKEAHA.123.044072 37485663 10.1161/STROKEAHA.123.044072 PMC11229702

